# An analysis of the spatio-temporal behavior of COVID-19 patients using activity trajectory data

**DOI:** 10.1016/j.heliyon.2023.e20681

**Published:** 2023-10-09

**Authors:** Xiumei Shen, Hao Yuan, Wenzhao Jia, Ying Li, Liang Zhao

**Affiliations:** a School of Architecture, Southeast University, Nanjing, 210019, Jiangsu, China; b AIM Architecture, Shanghai, 201100, China; c School of Energy and Environment, Hebei University of Engineering, Handan, 056038, Hebei, China

**Keywords:** Activity trajectory, Spatio-temporal behavior, COVID-19, Complex network, Community life circle

## Abstract

During the global pandemic, COVID-19 patients' activity trajectories and actions emerge as revelatory conduits elucidating their spatiotemporal behavior and transmission dynamics. This study analyzes COVID-19 patients' behavior in Nanjing and Yangzhou, China, by using patient activity trajectory data in conjunction with complex network theory. The main findings are as follows: (1) The evaluation of the activity network structure of patients revealed that “residential areas” and “vegetable markets” had the highest betweenness centrality, indicating that these are the primary nodes of COVID-19 transmission. (2) The power-law distribution of the degree distribution of nodes for different facility types revealed that residential areas, vegetable markets, and shopping malls had the most scale-free characteristics, indicating that a large number of patients visited these three facility types at a few access points. (3) Community detection showed that patient visitation sites in Nanjing and Yangzhou were divided into five or six communities, with the largest community containing the outbreak origin and several residential areas surrounding it. (4) Patients had fewer activities across administrative regions but more activities across the life circle when the pandemic broke out in the suburbs, and more activities across administrative regions but fewer activities across the life circle when the pandemic broke out in the central city. Based on these findings, this paper makes recommendations for future pandemic preparedness in an effort to achieve effective pandemic control and reduce the damage caused by pandemics. Overall, this study provides insights into understanding the transmission patterns of COVID-19 and may inform future pandemic control strategies.

## 1 Introduction

The COVID-19 pandemic has posed unprecedented challenges and had a tremendous impact on people's productive lives [1]. While many countries eventually adopted a policy of complete liberalization (i.e., the removal of all restrictions), some countries, such as China, implemented patient quarantine measures at the outset of the outbreak. The isolation and control measures in China proved to be a significant deterrent to the emergence and spread of the pandemic [2], significantly reducing the burden on global efforts to prevent and control the pandemic [3,4]. Building on this success, the activity trajectories of diagnosed patients are analyzed, and spatiotemporal comparative maps are created to identify potential exposure links between patients [5]. This information can then be used to adjust and optimize isolation control policies [6] to further control the spread of the virus.

Since the outbreak of the COVID-19 pandemic, geographic information systems (GIS) and spatial big data technologies have played a crucial role in containing the pandemic. As a result, numerous research projects using GIS for spatiotemporal analysis have emerged. These studies have included building an outbreak big data information system [5], using mobile data to explore spatial and temporal trends in COVID-19 transmission over time [7,8], predicting or simulating COVID-19 transmission [9,10], examining the association between COVID-19 transmission and other variables [11,12], and assessing the current status and supply-demand balance of health resources and health care delivery systems [13,14]. Through geospatial analysis, these studies have assessed the dynamics and trends of the COVID-19 pandemic and provided varying degrees of support for pandemic prevention and control, as well as the deployment and transportation of pandemic preparedness items.

Human spatiotemporal behavior can reflect the mobility of residents within cities, as well as the form and function of areas within cities, the location of facilities, and transportation needs [15], and can help to understand urban dynamics and elucidate the social factors that drive them [16]. The spatio-temporal behavior of COVID-19 patients prior to diagnosis can provide insight into both the spatial patterns of urban residents’ daily activities and the characteristics of viral transmission in the city. Previous studies of spatio-temporal behavior have often relied on activity logs, interviews, and other methods [17], but in recent years, an increasing number of studies have used GPS location data for exploration, such as having experimenters wear GPS trackers [15], using taxi track data (essentially also using the GPS location function of taxis) [16], and using GPS location data from open websites [18,19]. Regardless of the type of data used, the goal is to reveal the spatio-temporal patterns and drivers of human mobility behavior in order to achieve behavior management and adjustments to urban spatial structure.

In the context of a COVID-19 pandemic, it is essential to identify and understand the types of facilities that contribute to the transmission of the virus, in addition to focusing on population mobility. Previous studies [6,20] have identified restaurants, bars, fitness centers, hotels, and cafes as COVID-19 infection hotspots. A study conducted in the metropolitan area of Osaka [21] revealed that all generations visit groceries/pharmacies daily between March 2020 and September 2021 compared to other human mobility; therefore, it is necessary to control the mobility of people in groceries/pharmacies between −5% and 5 %. Another analysis using Twitter data [22] showed that during the entire COVID-19 blockade, Kuwaiti residents went to the suburbs and hospitals significantly more than usual, while they visited shopping malls significantly less. It is obvious that differences in urban structure, resident behavior, and management policies between countries and regions have led to differences in COVID-19 transmission facilities. Therefore, this paper intends to investigate the spatiotemporal behavior and potential infection facilities of COVID-19 patients prior to diagnosis under China's dynamic clearance policy, which is one of the innovations of this paper.

Although there are many studies using GIS to analyze the trend of COVID-19 transmission expansion [2], most of these studies focus on nationwide or intercity population mobility with large study scales and low precision. However, analyzing and comparing the spatial activity trajectories and spatiotemporal behavior of patients is an essential technical task for virus tracing and transmission chain reconstruction. Comparison of patient and population activity trajectories provides a crucial scientific basis for identifying groups at risk of infection [5,23]. As more specific transmission patterns are discovered, the significance of small-scale, precise trajectory analysis increases. In summary, this paper aims to determine the relationship between the spread of COVID-19 in cities and the activities of residents through detailed analysis and spatial mapping of patient activity trajectories and behaviors in the week prior to diagnosis. Using GIS and spatial big data technologies, we will analyze COVID-19 patients' spatiotemporal behavior to gain insights into the spatial patterns of daily activities and potential infection sites in the city. The findings will not only provide valuable and targeted suggestions for future epidemic prevention and control strategies but also help to adjust and optimize the spatial structure of the city and improve its ability to resist pandemics. Ultimately, this study will greatly contribute to urban outbreak control efforts and help reduce the impact of possible future pandemics on people's work and lives.

## 2 Materials and methods

### 2.1 Data sources

#### 2.1.1 Data generation

On July 20, 2021, some of the routine nucleic acid test samples collected from employees at Lukou International Airport in Nanjing, China, returned positive results [24]. Relevant personnel were isolated promptly, but because the outbreak was not immediately detected, the virus spread throughout the population. A viral carrier moved from Nanjing to Yangzhou on July 21 to visit relatives and spread the virus in Yangzhou [25]. The outbreak was finally brought under control thanks to the efforts of multiple parties. Before and after the outbreak, a total of 235 people in Nanjing and 570 people in Yangzhou were infected with the Delta virus.

Nanjing and Yangzhou were chosen as the study areas for two main reasons. First, the viruses in Nanjing and Yangzhou are homologous, belonging to the same transmission strain, which helps to exclude the influence of the virus's transmission ability on the results. Second, the outbreak sites of the virus in Nanjing and Yangzhou are significantly different, with one situated in the central city and the other in the suburbs. This distinction allows us to conduct a comparative analysis and reveal the transmission characteristics of the pandemic in different regions.

#### 2.1.2 Data collection

After the outbreak, the official websites of the Nanjing Health and Wellness Commission [26] and the Yangzhou Health and Wellness Commission [27] promptly posted text descriptions of the activities of confirmed patients. The data used in this paper were obtained from this source. The data in this study were published with the informed consent of the patients, under the premise of protecting individual privacy. Before publishing the data, the Health and Wellness Commission de-identified the information to ensure the complete anonymity of personal identities. The data were collected based on the patients' verbal descriptions, which included the sequence of daily locations visited but excluded the specific times and durations of stay at each location.

As well, the road network data were obtained from OpenStreetMap [28], and the water area data were obtained from the National Catalog Service for Geographic Information [29], all of which have been processed by ArcMap 10.6.

### 2.2 Data preprocessing

Considering the complex data structure of the trajectory text information, we manually extracted the daily visit sequence for each location based on the text describing the patient's daily itinerary and stored it in a structured Excel table. We also collected each patient's daily visit locations in chronological order and obtained their WGS-84 geographic coordinates using a Python-based web crawler, which leveraged the requests package to extract data from Google Maps. Since this study focuses on the in-depth analysis of patients' spatial movement trajectories, addresses without precise geographic coordinates cannot provide a comprehensive basis for our analysis and may introduce inaccuracies. Therefore, prior to storing the data in the final database, we implemented rules for data cleaning. The rules include: 1. removing locations with unidentifiable addresses, such as “a friend's house” and “a relative's house”; 2. removing patients who had only a confirmed diagnosis and isolation date but no detailed trajectories. By applying these rules, a total of 132 patients in Nanjing and 564 patients in Yangzhou with their detailed trajectory information were obtained. To analyze the spatial characteristics of behavior, all visit points were categorized based on the classification of facility types in the “Technical Guidelines for Community Life Circle Planning” [30].

#### 2.2.1 Complex network analysis

Complex networks can abstractly describe the movement behavior of patients between destinations, allowing researchers to better understand the spatial pattern of patient flow. Thus, the complex network theory was incorporated into the study of infectious disease transmission [31], providing a new view for the analysis of the spatial behavior of confirmed patients. In this paper's complex network, the locations visited by patients are represented by nodes, while the flow of patients between locations is represented by weighted edges. The weights are calculated based on the number of times a patient has moved between two locations. Specifically, structural evaluation and community detection are used to explore the network effects of patients' spatial behavior. In this paper, the transmission network is analyzed and visualized using Gephi 0.9.7. Gephi 0.9.7 is an open-source, free visualization and exploration software designed to work with various types of graphs and networks [32]. It has been widely used in fields such as social network analysis [33].

● Structural Evaluation

Networks can be analyzed quantitatively using statistical indicators such as degree, modularity, and centrality [34]. In this study, five indicators, weighted degree, weighted in-degree, weighted out-degree, betweenness centrality, and closeness centrality, were chosen to evaluate the structural characteristics of the patient flow network at each node (Table 1). The indicators were calculated using Gephi 0.9.7.

**Table 1:** The applied indicators of network structure evaluation.

Name	Definition	Formula
Node degree	Reflects the importance of nodes in the network.	$d_{i}=\sum_{j=1}^{n}L_{ij}$ Where, *Lij* is the number of edges between node *vi* and node *vj*; *n* is the total number of nodes.
Weighted degree	Reflects the connection frequency between the target node and the adjacent node in the network, further divided into weighted in-degree and weighted out-degree.	$S_{i,out}=\sum_{j\in N_{i}}w_{i,j}$ $S_{i,in}=\sum_{j\in N_{i}}w_{i,j}$ $\bar{S}=\frac{\sum_{i-1}^{n}S_{i,in}+S_{i,out}}{2n}$ Where, *Si, out* and *Si, in*, are weighted in-degree and weighted out-degree respectively; *Ni* is the set of adjacent points of node *vi*; *wij* is the weight of the directed edge from node *vi* to node *vj*, i.e., the number of tourists; $\bar{S}$ is the average weighted degree of the whole network; *n* is the total number of nodes.
Weighted in-degree	Weighted in-degree means number of inbound links toward a node.	
Weighted out-degree	Weighted out-degree means number of outbound links from a node.	
Betweenness centrality	Betweenness centrality reflects the degree of control (power) of the target node on other nodes in the network.	$BC\left(v\right)=\sum \frac{{pd}_{st}\left(v\right)}{d_{st}}$ Among them, d_st_ represents the number of shortest paths from s to t, and pd_st_ represents the number of shortest paths passing through the given node v from s to t.
Closeness centrality	Closeness centrality quantifies the average distance between a node and every other node in a network. A node with a high closeness centrality has a minimal average distance from every other node in the network, indicating that it is highly connected and plays a central role in the network.	The closeness centrality CC_i_ of a node is:$d_{i}=\frac{1}{n-1}\sum_{j\neq i}d_{ij}$ ${cc}_{i}=\frac{1}{d_{i}}=\frac{n-1}{\sum_{j\neq i}d_{ij}}$ Where d_i_ represents the average distance from the node to the rest of the points, and the reciprocal of the average distance is the closeness centrality.

The node's weighted degree indicates the total frequency with which all patients visit the node. The greater the weighted degree of the node, the greater the number of patients visiting the activity site and the greater the risk of infection at the site. The weighted in-degree of a node is the total frequency of reaching the node, and evaluating the weighted in-degree helps to understand the events that lead to node infection. The weighted out-degree of a node is the frequency of all outbound connections. The higher the out-degree of a node, the greater its impact on the pandemic and the more severe the consequences. And the greater the number of such nodes, the easier it is for the virus to spread and the more rapidly the number of confirmed cases will rise [35]. Higher betweenness centrality nodes are more strongly associated with the subsequent infection [35]. In addition, closeness centrality is the metric most directly applicable to infectious disease research, as it quantifies the connection of nodes or their importance within the network [36].

● Degree distribution of nodes

The power-law relationship is a statistical rule that is widely used in complex networks. In the field of network science, power-law relationships are commonly used to describe the degree distribution of nodes in networks [37,38]. When a network's degree distribution satisfies the power-law relationship, it means that the probability distribution P(k) of the node degrees in the network is inversely proportional to the degree k, i.e., P(k) ∝ k^(-γ), where γ is known as the power-law exponent. The manifestation of power-law distribution in networks is that a few nodes have very high degrees of connectivity (i.e., “rich nodes”), while the vast majority of nodes have lower degrees of connectivity. Power-law relationships in network research can be used to identify and prioritize important nodes. In networks based on power-law relationships, highly concentrated nodes tend to play key roles [18]. By identifying these important nodes, community structures within the network can be discovered, as well as nodes with critical influences on network functions and stability. In the specific computations, we use Gephi 0.9.7 to calculate the degree of each node, followed by Python 3.0 to calculate the node degree distribution. Subsequently, SPSS 25 is utilized to assess whether the node degree distribution conforms to a power-law distribution.

● Community Detection

Communities in complex network theory are dense subnetworks within a larger network [39]. Each of these clusters or groups of nodes has significant internal cohesion (edge density within the group) but weak external cohesion (outside the group) [40]. Community detection is a classical method for understanding the spatial structure of complex networks in geographic space [41].

Among other community detection methods, the modularity-based algorithm has demonstrated superior performance [42], and modularity-based community detection can identify the partition with the largest modularity among all possible community partitions. Among them, the modified Louvain algorithm [43] is generally regarded as the best modularity-based community detection approach because the addition of a resolution parameter can change the number of communities detected. The lower the resolution, the more communities are discovered; the higher the resolution, the fewer communities are discovered. In this study, modularity was calculated using the Louvain algorithm. The Louvain algorithm has been integrated into Gephi [44], so we use Gephi 0.9.7 to detect the communities of patient visit sites.

#### 2.2.2 Travel range analysis

The range of resident activities was examined in two dimensions: the administrative area and the 15-min life circle. Patients who move from one administrative area to another may experience long-distance transmission that is difficult to manage. The 15-min walking life circle (about 1 km in distance) can efficiently meet the daily needs of residents under normal conditions, and the Chinese government has aggressively promoted its implementation [45]. Furthermore, a 15-min cycling circle (about 4 km) was also evaluated, based on the possibility that residents may need to travel further to meet their daily needs after the regional blockade.

By using the Isochone API [46] under mapbox and providing the geographic coordinates of the patient's residence, the Python program can batch obtain the boundary range polygons for walking and cycling within 15 min of the starting point. Import the residence, visit, and boundary polygons for each patient into Rhino 6. Evaluate the relationship between the visited points in the travel trajectory of confirmed patients, the administrative divisions, and the 15-min travel circle after counting and evaluating the proportion of visited points within the boundary polygon.

## 3 Results

### 3.1 Basic information statistics

#### 3.1.1 Patient's age

**Fig. 1:**
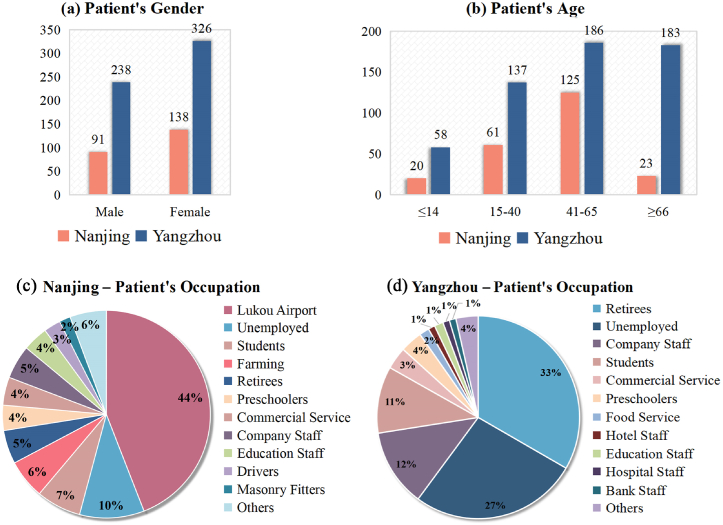
Basic information statistics of patients in two cities.

Fig. 1 (a, b) show the age and sex of the patients. Fig. 1a shows that although the number of infected individuals in the two cities is different, the proportion of females is greater than that of males. Since the majority of infected individuals in Nanjing are Lukou Airport employees, the middle-aged individuals are the largest. Yangzhou has a high proportion of middle-aged and elderly citizens. In both cities, children make up a small proportion of the affected population.

#### 3.1.2 Patient's occupation

In terms of patients’ occupations, besides the staff at Lukou Airport (the source of the outbreak), Nanjing City has the highest percentage of unemployed individuals, followed by students. In addition, due to the location of Lukou Airport in the suburbs of the city and the fact that most of its employees live in rural areas, a disproportionate number of those affected are farmers (Fig. 1c). In Yangzhou, the largest number of patients are retirees, followed by the unemployed, corporate employees, and students (Fig. 1d). Students, retirees, and unemployed individuals are the three largest groups affected by this outbreak in both cities.

### 3.2 Activity network analysis

#### 3.2.1 Network structure evaluation

**Fig. 2:**
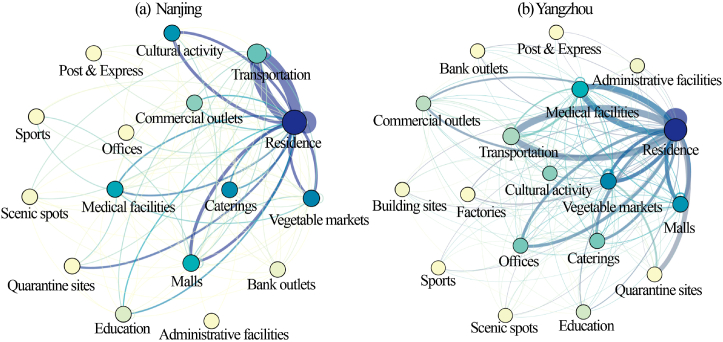
Network diagram of patient travel between sites.

Fig. 2a, b shows the patient travel network constructed between sites using trajectory data. The raw data captured the patients' movements from specific facilities, such as “Maoting Community” and “Fengxiang Community”, to other different destinations like “Four Seasons Vegetable Markets” and “Shengzhuang Vegetable Markets.” For ease of analysis, we generalized these specific facilities into their respective types, such as “Residence” or “Vegetable Markets”. Thus, within the network, each node represents a type of facility (e.g., “Residence” or “Vegetable Markets”), and the edges represent the movement of patients from one type of facility to another. The weighted degrees of residence nodes are the largest in both Nanjing and Yangzhou, with 1810 and 5261, respectively, and the weighted out-degree and weighted in-degree of residence are significantly higher than those of other node types. The most frequent travel was between the residence and the origin of the pandemic. It is between residences and Lukou Airport (a transportation facility) in Nanjing and between residences and chess and card rooms in Yangzhou (a cultural and activity facility). These two types of locations are “hubs” for patients' daily activities, as they have the highest betweenness centrality. Other facilities with high betweenness centrality include shopping malls, cultural and activity facilities.

Table 2 shows the more specific structural characteristics of the trajectory network in Nanjing and Yangzhou. It indicates that residences and vegetable markets have the highest closeness centrality, indicating that these two types of facilities are the main points of pandemic transmission. And because they linked to the largest number of nodes, it further shows the importance of their core nodes. Administrative facilities have the least weight and access, and patients visit them the least frequently. In addition, the close centrality of the administration is the lowest, showing that confirmed patients view the administration as a distinct place with the weakest relationship to other types of facilities.

**Table 2:** Patient trajectory network structure characteristic parameter table.

Facility types	Number of facilities	Weighted degree	Weighted indegree	Weighted outdegree	Betweeness centrality	Closness centrality
Residence	105, 258	1810, 5261	870, 2452	940, 2809	70.97, 63.81	0.94, 1.0
Vegetable markets	22, 79	239, 1238	119, 616	120, 622	19.73, 27.42	0.83, 0.89
Transportation facilities	17, 16	773, 142	392, 68	381, 74	5.23, 6.09	0.68, 0.74
Restaurants	29, 119	112, 611	56, 305	56, 306	16.87, 6.89	0.62, 0.74
Medical facilities	19, 39	145, 1027	71, 494	74, 533	10.32, 11.15	0.75, 0.75
Isolation facilities	1, 1	70, 442	68, 440	44, 959	0.0, 0.0	0.5, 0.53
Postal logistics express facilities	3, 3	14, 26	7, 13	7,13	0.0, 0.0	0.54, 0.53
Commercial outlets	22, 53	70, 346	35, 173	35,173	4.15, 3.54	0.6, 0.71
Shopping malls	40, 91	211, 840	105, 418	106, 422	7.83, 21.56	0.65, 0.85
Sports facilities	4, 9	30, 42	15, 21	15, 21	0.0, 0.27	0.54, 0.57
Cultural and activities facilities	21, 43	152, 1465	76, 722	76, 743	14.98, 4.37	0.65, 0.71
Office	5, 50	18, 510	9, 253	9, 257	0.0, 6.98	0.6, 0.74
Scenic spots	4, 11	31, 40	15, 20	16, 20	0.17, 0.22	0.58, 0.61
Educational facilities	15, 29	82, 148	41, 74	41, 74	1.18, 2.21	0.62, 0.65
Bank branches	4, 5	31, 66	15, 33	16, 33	0.57, 0.0	0.62, 0.59
Administrative facilities	1, 10	2, 46	1, 23	1, 23	0.0, 0.49	0.42, 0.61

Note: The left side of "," is the data of Nanjing, and the right side is the data of Yangzhou.

**Fig. 3:**
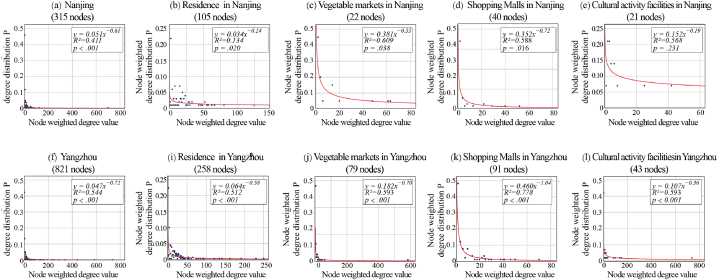
Node degree distribution curves of various facilities in the trajectory network.

Recall that in our network, a ‘node’ corresponds to a facility type, and an ‘edge’ represents the movement of patients between these facility types. The degree of each facility type node in SPSS 25 is modeled using a power law distribution, and the results are shown in Fig. 3a-l. It shows that the network of patient trajectory points is very heterogeneous and scale-free; that is, nodes with a higher weighting degree are fewer in number. Although the fitted curve of the degree distribution is modified by the number of nodes in the network, this effect can be ignored when comparing networks with similar (or comparable) numbers of nodes [18]. Thus, the R^2^ value allows quantitative comparisons of the scale-free characteristics of each facility's access network (i.e., power of fit). We chose the four most influential node types—residence, vegetable markets, shopping malls, and cultural activity facilities—and examined the distribution of visit points for each facility type.

In both Nanjing and Yangzhou, residences, vegetable markets, and shopping malls have the most visible scale-free characteristics. This indicates that, despite the enormous number of patients visiting these three types of facilities, the visit points are concentrated in a small number of sites. This is particularly noticeable in vegetable markets and shopping malls (the R^2^ of both types of facilities is higher than that of the residence). Patients' visits to Yangzhou's cultural activities facilities also showed scale-free characteristics. However, the significance of P > 0.1 in the power-law distribution fit in Nanjing indicates that its weighted degree distribution does not satisfy the power-law distribution and shows weak scale-free characteristics. This means that the distribution of patient visits to various cultural activity facilities in Nanjing is pretty even.

#### 3.2.2 Community detection

**Fig. 4:**
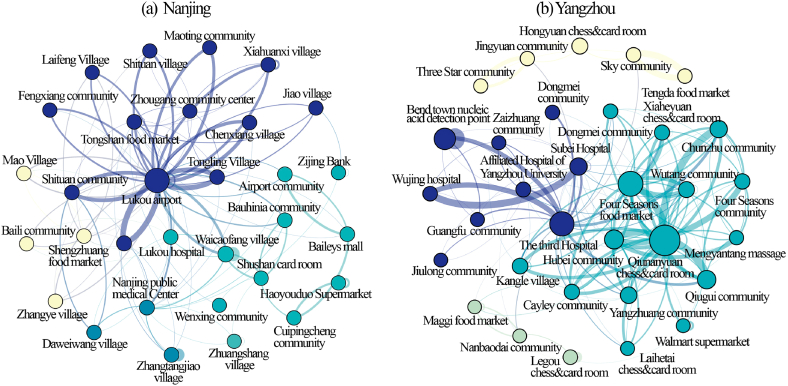
Visualization of community detection results.

A modified Louvain algorithm was employed to identify communities in the network of patient visits. To improve the visibility of the network, the facility nodes with the most recent 30 weighted degrees are filtered out. The Louvain algorithm resolution in Gephi is set to the default value of 1.0. Fig. 4a, b shows the results, and each community has a specific color.

The visit nodes in Nanjing are grouped into five communities. The largest community comprised over half (46.67%) of the nodes, which included Lukou Airport, many nearby residential communities, and a vegetable market. The second-largest community (16.67% of the nodes) has community hospitals and chess and card rooms. The isolation point and many nearby residential areas form the core of the third-largest community. The remaining two communities have fewer nodes, but both consist of residential areas and commercial facilities (supermarkets and shopping malls).

The patient visit facilities in Yangzhou are divided into six communities. The largest community was made up of 57.58 percent of all nodes; it includes the infection source (the Chunan Chess Room) and several nearby residential areas. Medical facility nodes make up the second-largest community; many major COVID-19-designated hospitals, nucleic acid testing sites, and centralized isolation points constitute the primary nodes of this community. Other communities are similar to Nanjing, consisting of a central commercial facility (such as vegetable markets or chess and card rooms) and several outlying residential communities.

### 3.3 Activity space analysis

#### 3.3.1 Distribution and timing of activity locations

**Fig. 5:**
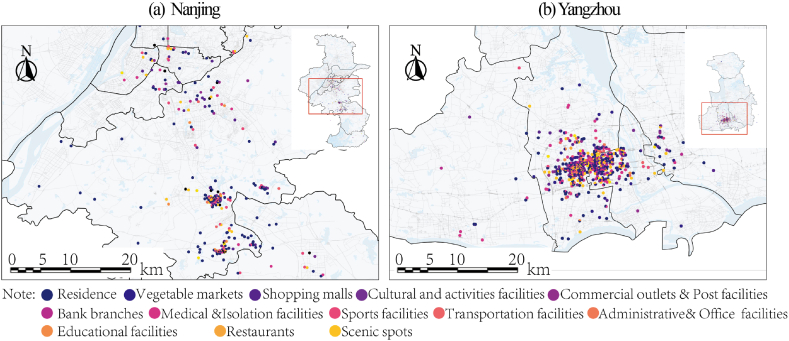
The locations of each type of facilities.

**Fig. 6:**
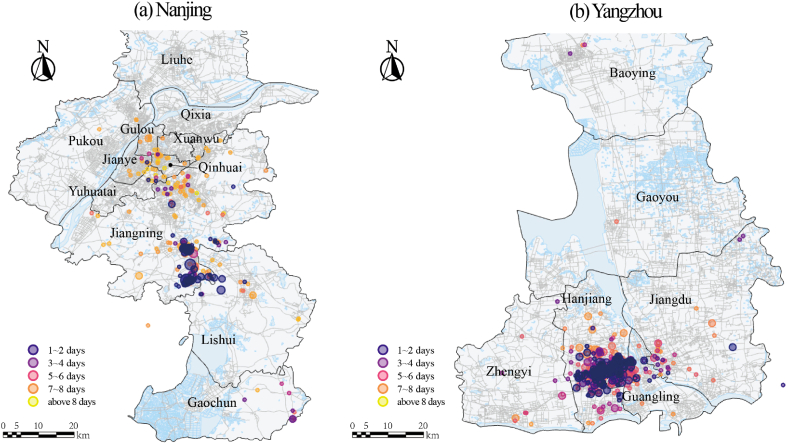
Sites and timing of patient visits.

Fig. 5a, b shows the locations of various types of facilities visited by the patients, while Fig. 6a, b further illustrates the sites and timing of patient visits. The sites of patient visits in Nanjing formed two geographically distinct spread hubs. The first spreading hub is located near Lukou Airport, where the outbreak began, in the city's suburbs. During the first one to two days of the outbreak, the majority of patients stayed within a one-to two-mile radius of the epicenter. The visit sites gradually spread throughout the central urban areas (Gulou and Qinhuai District) five days after the outbreak, forming a second spread hub. However, the visitation sites in Yangzhou have only one spreading hub, which is located near the Qiuyuan Chess and Card Room in the Hanjiang District, the center of the city. And within three to four days, it rapidly spread to the surrounding area, producing a gathering place in the city's central districts of Hanjiang and Guangling.

**Fig. 7:**
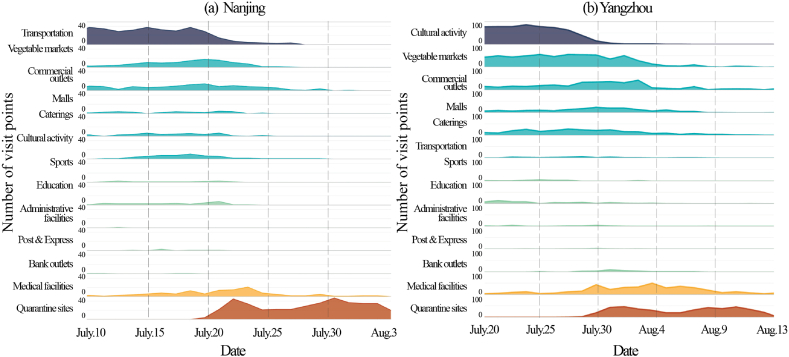
A trend graph of the types of facilities visited by patients over time.

Fig. 7(a, b) shows the trend in the type of facility visited by patients over time. According to the trend, the facilities can be loosely classified into four categories: the first is the source of the outbreak, and visits to these facilities peaked quickly and then declined rapidly (purple in the figure). This indicates that the outbreaks at Nanjing Lukou Airport (a transportation facility) and Yangzhou Chess and Card Room (a cultural facility) were quickly and efficiently controlled due to the effective measures. The second category consists of residences and other living facilities (shopping malls, commercial outlets, vegetable markets, etc.) (blue in the figure). After the visits to infectious sources began to decline, the visits to these facilities continued to increase. During this period, the virus spreads from its source to the residential communities. The third category contains medical facilities. Since the beginning of symptoms in infected patients, the need for medical treatment and nucleic acid testing has led to an increase in visits to medical facilities during this time period (orange in the figure). The fourth category is isolation facilities, indicating that the pandemic is being gradually controlled and patients are being treated in these facilities (red in the figure).

#### 3.3.2 Range and characteristics of activities

**Fig. 8:**
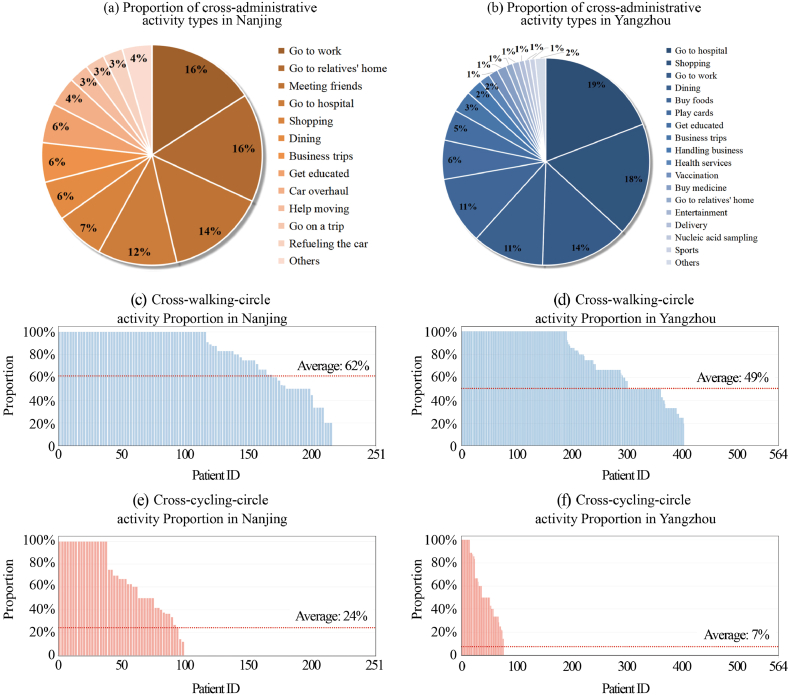
Proportion of cross-life circle and cross-administrative activity types in Nanjing and Yangzhou.

When activities need to be performed across administrative regions, the situations in the two cities are different. Twenty-six patients traveled across administrative regions in Nanjing (19.7 % of the samples). And there were 171 patients in Yangzhou who traveled across administrative regions (30.3 % of the samples). In general, the cross-administrative activities of Yangzhou patients were larger than those of Nanjing. In Nanjing, patients' cross-administrative activities mainly consist of going to work, visiting relatives, meeting friends, going to hospital, and shopping (Fig. 8a). And Yangzhou patients go to hospital, followed by shopping, going to work, dining out, and buying food (Fig. 8b). Combining the two cities, going to work, going to the hospital, and going shopping are the three most common cross-administrative activities.

The ratio of the number of places outside the life circle visited by each patient to the total number of sites visited by each patient in Nanjing and Yangzhou was calculated (Fig. 8a–d). In general, the number of patients visiting sites outside their life circle was higher in Nanjing (where the outbreak originated in the suburbs) than in Yangzhou (where the outbreak originated in the city center). And so, the activity sites outside the 15-min walking circle are larger than those sites outside the cycling circle. In particular, the percentages of visits to locations outside the life circle of Nanjing patients who cycled and walked for 15 min were 62 % and 24 %, respectively (Fig. 8c, e), which were both higher than the 49 % and 7 % for Yangzhou patients (Fig. 8d, f).

## 4 Discussion

### 4.1 Temporal and spatial behavioral characteristics of patients

Despite the fact that the outbreaks in the two cities originate from the same source, the Nanjing outbreak is located at Lukou Airport in the city's suburbs, is surrounded by a large area of farmland, and is spreading slowly. In Yangzhou City, on the other hand, the pandemic started in the central city, where there are many people. And the first transmission happened in a closed room where people played chess and cards, which caused the number of cases to rise rapidly. Numerous previous studies also demonstrate that high population density, low green space density, and high building density are associated with a high prevalence of COVID-19 [12,47].

Over time, the pandemic transmission center in Nanjing has moved from the Jiangning District suburbs to the city center (Xuanwu District, Qinhuai District). On the one hand, this is a result of the separation of employment and residence in urban areas. Virus carriers who live in the suburbs have to travel to the city center for work, resulting in the urban spread of the virus. On the other hand, the support facilities in the urban area are superior to those in the suburbs, and people from the suburbs must travel to the center more frequently. In contrast, the transmission center in Yangzhou was in the urban area because urban residents have less need to travel to the city's suburbs.

Further, the range of patients' activities varied. The proportion of Nanjing patients' activities across-administrative regions is relatively low, and most of their activities occur within the administrative region where the pandemic originated (Jiangning District). About half of the small number of cross-regional activities are non-daily activities such as shopping in the city's bustling commercial district (Xinjiekou) and visiting friends and relatives. When an outbreak occurs, these behaviors can be reduced through intervention, allowing for regional control based on administrative boundaries. It is worth noting that tertiary hospitals in the city center are another major factor in cross-administrative travel. For serious illnesses, Chinese patients prefer to seek medical care in large hospitals with a good reputation. An influx of patients has spread the virus to the densely populated urban center. When implementing control measures, the needs of these patients can be met by opening medical channels or by sending personnel and materials from large hospitals to community hospitals where the pandemic is developing.

The proportion of Yangzhou patients who travel across administrative regions is relatively high. This may be due to the fact that when the source of infection is located in the central metropolitan area, the transportation network is relatively well established, and individuals can easily use subways, buses, and other modes of transportation to travel to other administrative districts. It's also due to the fact that living facilities in the city center are relatively abundant; the majority of the activities of patients take place within a 15-min cycling circle. Therefore, if a pandemic breaks out in the central city, it is possible to control it based on the life circle.

Regarding the types of locations, initially, during the pandemic's onset, the areas most frequently visited by patients were located at the source of the pandemic. However, as time passed and the pandemic's source was controlled, the majority of patient locations were concentrated in residential neighborhoods. Within these neighborhoods, vegetable markets and cultural facilities (especially chess and card rooms) were the main areas where residents congregated. As the virus spread within residential areas, many residents with symptoms sought medical help at hospitals, making medical facilities an important entry point for a significant number of patients. This differs from findings in other countries. Previous research in Japan [21] found that groceries/pharmacies and parks were places of high mobility and that reducing the mobility of people in these places was necessary. However, a multinational study [48] with data from several countries, including the EU and the US, showed that changes in mobility in places such as restaurants, cafes, grocery stores, transit stations, and parks played a more important role in reducing disease transmission than in workplaces or residential areas. Data from Florida [49] also showed that the increase in average visits to bars and restaurants was one of the main factors contributing to the increase in COVID-19 cases. There may be two main reasons for these differences. First, during the early stages of the COVID-19 pandemic, China implemented a strict “zero-tolerance” policy [50], which included mandatory mask-wearing in public places, widespread nucleic acid testing, and other measures. As a result, viral transmission occurred mainly at the origin of the outbreak and in residential communities, while transmission in other public places was relatively low. In contrast, other countries implemented relatively relaxed policies, resulting in different transmission patterns in public places. Second, the proportion of middle-aged and older people in the sample studied in this article was relatively high. In China, this group tends to gather in places like chess rooms, while in the United States, bars and restaurants are more popular among younger people, making them important places for COVID-19 transmission.

### 4.2 Implications for practice

Based on the research findings, the following suggestions for future pandemic prevention and control in the event of a major pandemic were proposed:

(1) After the outbreak of the pandemic, China implemented measures of partial regional lockdown and nucleic acid testing for all individuals, which quickly interrupted the transmission chain of the virus. Therefore, it is necessary to appropriately restrict and guide the behavior of residents during the pandemic. However, when determining the range of guidance and restrictions, it is important to consider the geographic location of the outbreak. If the outbreak occurs in the suburbs, administrative boundaries are more appropriate, whereas if the outbreak occurs in the city center, the use of life circles would be more appropriate. This paper found that life circles more realistically reflect pandemic transmission in central urban areas. Utilizing the life circle's range, especially the 15-min cycling circle, it is possible to split the pandemic's afflicted areas more precisely and take more effective measures to stop its spread.
(2) Second, this study found that going to work, going to the hospital, and shopping are the three most common types of cross-administrative activities. Therefore, in the event of a pandemic, residents should be encouraged (and, if necessary, required) to adopt online work from home, online shopping, and online consultation whenever possible.
(3) Third, besides controlling the source of transmission, it is vital to pay particular attention to the communities where the patients live, as well as to vegetable markets, shopping malls, and other locations with high population turnover; improve the management and inspection of these locations. In addition, the testing and management of medical facilities must be improved.
(4) Fourth, this study indicated that residents' everyday activities are relatively fixed. Hence, using public surveys, it is possible to discover the most frequented sites, and control should be prioritized there. However, public surveys alone cannot manage the pandemic. Other elements, including the disease's spread, medical resources, and economic development, must be considered to produce scientific and appropriate policies and measures.
(5) Considering the large area and significant separation between work and residence in Nanjing, cross-administrative commuting and medical visits are common, which could lead to long-distance transmission in the event of an outbreak. When an outbreak occurs, it is recommended to strengthen remote work and online medical consultations while enhancing collaboration among different regions. Relevant departments and medical institutions should promptly share information and allocate resources to enhance pandemic control capabilities across a broader scope. Additionally, implementing staggered commuting and transportation management is advised to mitigate transmission risks during the commuting process.
(6) In the case of Yangzhou, the majority of this outbreak's transmission was linked to enclosed spaces like chess and card rooms, where a significant proportion of the city's elderly population tends to gather for entertainment. Therefore, stringent control over indoor population density in public spaces is essential during an outbreak. Adequate ventilation through opening windows is crucial. Simultaneously, reinforcing community patrols and pandemic awareness campaigns can guide the elderly to stay at home and reduce unnecessary outings.

### 4.3 Limitations and directions for future research

Although we have conducted a more thorough analysis, this paper has a few limitations. The data in this paper were obtained from the National Health Commission's patient trajectory text data, and the data's temporal precision is poor. Only the order of visits to the patients' main daily activities and minimal information on the patient's mode of transportation are reported; therefore, the analysis of temporal behavior and mode of transportation is limited. Furthermore, the trajectory of each patient is based on the patient's own oral recollection, without specific information about the exact time and duration of each location stay. This also leads to biases in estimating the probability of different facilities becoming sources of infection. If future research is able to combine cell phone GPS positioning data, the research's accuracy will be substantially improved.

Due to the limited sample size, the study only analyzes the trajectory of confirmed patients in Nanjing and Yangzhou. Also, the spread and growth of the pandemic are random and unpredictable, our analysis and findings may also be randomized. In the future, if a more extensive insight into the mechanism of the spread of the pandemic is required, data from additional regions can be collected for comparative research in order to undertake a more thorough and objective analysis of the spread and early development of the pandemic.

## 5 Conclusions

This paper focuses on the activity space and mobile network of COVID-19 patients prior to their diagnosis, and attempts to identify which facilities in the city are potential transmission sources of the COVID-19 epidemic and which resident behaviors are more likely to cause the virus to spread in the city under the dynamic “zero tolerance” measures implemented in China. We constructed a database using textual information on the trajectories of confirmed patients published on the Health Commission's official website. Combined with GIS, Gephi, Python, and other applications, the spatial trajectory and spatiotemporal behavior of the patient can be detected and analyzed in depth. The key findings can be summarized as follows:

(1) Among these confirmed patients, the middle-aged individuals were the largest, the children were the smallest, and the number of women was somewhat greater than that of men. Students, retirees, and unemployed individuals are the three largest groups in this spread.
(2) Patients move to many different places during an outbreak, besides their homes and the place where the disease started. However, they most frequently go to vegetable markets, shopping malls, and medical facilities.
(3) The weighted distribution of nodes indicates that people's activities are regular. Even though there are various types of places for people's daily activities, the exact sites for these activities are rather fixed.
(4) There are variances in the types of cross-regional activities of patients in different cities. When the pandemic's origin is in the suburbs, patients engage in fewer cross-administrative activities and more cross-life circle activities, whereas when the pandemic's origin is in the city center, patients engage in more cross-administrative activities and fewer cross-life circle activities. In general, going to work, going to the hospital, and shopping are the three most common cross-administrative activities for patients.
(5) As a result of the urban-rural layout, the suburban population density is relatively low, people engage in a wide range of activities, and the transmission of the virus had polycentric characteristics. In contrast, the central urban area has a relatively high population density and a compact life circle, so the spread of the infection has the features of a single center.
(6) From the perspective of site type, the chronological order of hotspots for patient visits is as follows: the origin of the pandemic, residential service facilities, medical facilities, and isolation facilities.

## Funding

This research received no external funding.

## Data availability statement

The datasets generated during and/or analyzed during the current study are accessible in a publicly available repository. The data-sharing website is: https://doi.org/10.6084/m9.figshare.22188742.v1.

## Ethics declarations

Review and/or approval by an ethics committee was not needed for this study because the data used in this paper are publicly available.

## CRediT authorship contribution statement

**Xiumei Shen:** Conceptualization, Data curation, Investigation, Methodology, Project administration, Software, Validation, Visualization, Writing – original draft. **Hao Yuan:** Conceptualization, Data curation, Methodology, Software, Validation, Visualization, Writing – original draft. **Wenzhao Jia:** Data curation, Resources, Supervision, Writing – review & editing. **Ying Li:** Project administration. **liang Zhao:** Data curation, Supervision.

## Declaration of competing interest

The authors declare that they have no known competing financial interests or personal relationships that could have appeared to influence the work reported in this paper.
